# Association between psychological distress and coping strategies among students engaged in online learning

**DOI:** 10.1371/journal.pone.0270877

**Published:** 2022-07-01

**Authors:** Nusrat-E- Mozid

**Affiliations:** 1 Department of Public Health, North South University, Dhaka, Bangladesh; 2 Public Health Professional Development Society (PPDS), Dhaka, Bangladesh; Sapienza, University of Rome, ITALY

## Abstract

Distant or online learning on digital platforms has become the norm in education worldwide, putting students under immense mental strain. The present study examined the association between psychological distress and coping strategies among students engaged in online learning. This study used a cross-sectional design. A structured questionnaire was sent to each of the 210 university students at two prestigious public and private universities in Bangladesh through email. Data was collected from March 26 to April 27, 2021. Severe psychological distress was found in 55.2 percent of the population. Younger age groups, public university students, students with no self-income, moderate aid from the university in providing resources from home, and students with existing mental health illnesses were significantly associated with psychological distress (*p*-value<0.05). Among all 14 items of Brief COPE, active coping, using emotional support, and self-blame significantly influenced the psychological distress instrument Kessler-10 (*p*-value<0.01). Given the perspective of this study, coping strategies alleviate stress and facilitate positive psychological outcomes. Students’ mental health is a prioritized issue that needs more attention. Because of its higher prevalence and adverse consequences, institution authorities should support each student by providing study materials, student loans, and scholarships. Routine screening will allow identifying students going through a difficult time who can get help from experts through counseling.

## Background

The COVID-19 pandemic has interrupted many facets of everyday life, with some of its greatest impacts seen in the education sector [1,2]. Approximately half of students worldwide have faced the closure of schools, colleges, and related institutions. Therefore, the continuation of the education process with remote or online learning on digital platforms was necessary to avoid a generation gap [3]. The adoption of this new delivery model of education appeared simple and has proven to be beneficial in several aspects. However, the evolution of the virus and the resulting social isolation caused significant mental health problems among many students [4]. Early on, the American Psychological Association foreshadowed that coronavirus would have far-reaching consequences, particularly in terms of mental stability [5]. Students’ increased emotional pressures nowadays trigger various diseases and deteriorate their psychological health [6]. A heightened risk of experiencing stress, depression, anxiety, and suicide risk behaviors has been reported, particularly among university graduates [7]. Although students report the highest stress levels, they have received little attention. Apart from the traditionally lengthy study hours and heavy course loads, university students encounter various challenges. Certain factors, such as financial stress, fear of career uncertainty, staying long hours at home, and loneliness, have all been identified as major stressors during the pandemic [8].

Psychological distress is indicative of impaired mental health, and it contributes to a slew of other disorders [9]. Chronic immune activation and adverse health outcomes are caused by stress. People who experience high-stress levels manifest exacerbated symptoms of both physical and psychological illnesses [10]. These consequences have shown elevated prevalence rates during the COVID-19 pandemic. An online survey conducted in Austria showed that depression and anxiety symptoms were higher in younger age groups and those with no income [11]. Studies also revealed that COVID-19-related emotional and social outcomes among students were linked with travel restrictions, home isolation, and fear of coronavirus infection [12,13]. Likewise, a study from Bangladesh found that 37.7% of university students experienced severe stress, which led to insomnia, irritability, and poor academic performance [14].

The education system in Bangladesh was unprepared to deal with the pandemic, with few alternate arrangements and facilities in place for efficient teaching and learning [15]. Home quarantine amid the lockdown induced uncertainty in academia [16]. A study on tertiary-level Bangladeshi students’ online class experiences showed less preparedness for online classes, lower attendance, and fewer class activities [17]. Many students found online assessments confusing, and poor internet bandwidth during exams was commonplace [18]. A lack of regular contact and monitoring by university personnel has led to social isolation, impaired executive function, and cognitive decline [19]. According to a cross-sectional study of university students in Bangladesh, 58 percent of students were at a sub-optimum level of e-learning readiness and suffered moderate to high-stress levels [20]. However, in today’s technological era, innovative online learning is no longer a pipe dream but rather one of the standard modalities of higher education [21]. A study exploring online learning experiences for sub-degree students in Hong Kong displayed that network speed plays a crucial role in strengthening students’ potential for in-depth and purposeful learning [22]. Students with self-directed learning skills, technology readiness, and motivation, on the other hand, can more readily attain critical online learning skills [23]. Institutions can also play a vital role by training prospective teachers to help students transition smoothly during challenges like COVID-19 [24].

Coping strategies are actions, a series of behavioral and cognitive tactics used to deal with upsetting situations, conditions, and demands [25]. The COPE has been a commonly used tool in health psychology research. Different models of coping strategies have been established and measured [26]. However, the interventions’ tools and their effectiveness vary across the literature. Problem-focused and emotion-focused coping were identified by Lazarus and Folkman (1984), but the distinction between these two outcomes was not conceptually evident [27]. Coping dimensions theoretically derived by Carver and colleagues worked on the difference and found it insufficient. They identified 13 dimensions with two items each. Problem-focused coping with five sub-dimensions, emotion-focused coping with another five sub-dimensions, and the remaining three were classified as ‘less useful’ strategies, with humor and substance use as additional items [28]. Most recently, Cooper and colleagues classified the original subscales of brief-COPE [29] into three categories- a) problem-focused (active coping, planning, and use of instrumental support), b) emotion-focused strategies (use of emotional support, positive reframing, acceptance, religion, and humor), and c) dysfunctional or avoidance (venting, denial, substance use, behavioral disengagement, self-distraction, and self-blame) [30]. This study utilized the behavioral self-regulation model of Carver, the brief COPE, because of its excellent psychometric properties and wide variety of use.

### The present study

Everyone experiences stress to some extent, and university students are no exception. Academic life can be stressful for many students, with environmental, social, or internal demands that cause them to adjust their behavior. However, this stress does not always take a negative form. Research shows that positive stress can enhance memory and improve risk-taking and decision-making skills [31]. While there is no perfect way to control stress, using effective techniques can help during stressful times and turn students’ academic, social, and emotional circumstances into more positive and successful experiences. When applied to the COVID-19 pandemic, these techniques can limit the adverse effects of online learning, including changes in eating and sleeping patterns, separation from classmates, and loneliness. Therefore, the present study aims to ascertain the association between psychological distress and coping strategies among students engaged in online learning.

### Research questions

This study tried to answer the following research questions:

1. What is the prevalence of psychological distress among university students engaged in online learning during the COVID-19 pandemic?
2. Is there any association between psychological distress and students’ socio-demographic characteristics?
3. Is there any association between psychological distress and coping strategies among students engaged in online learning?
4. Which coping strategy has primarily been used in managing stressful events by students occupied with distance learning?

## Methods and materials

### Participants and procedures

**Fig 1:**
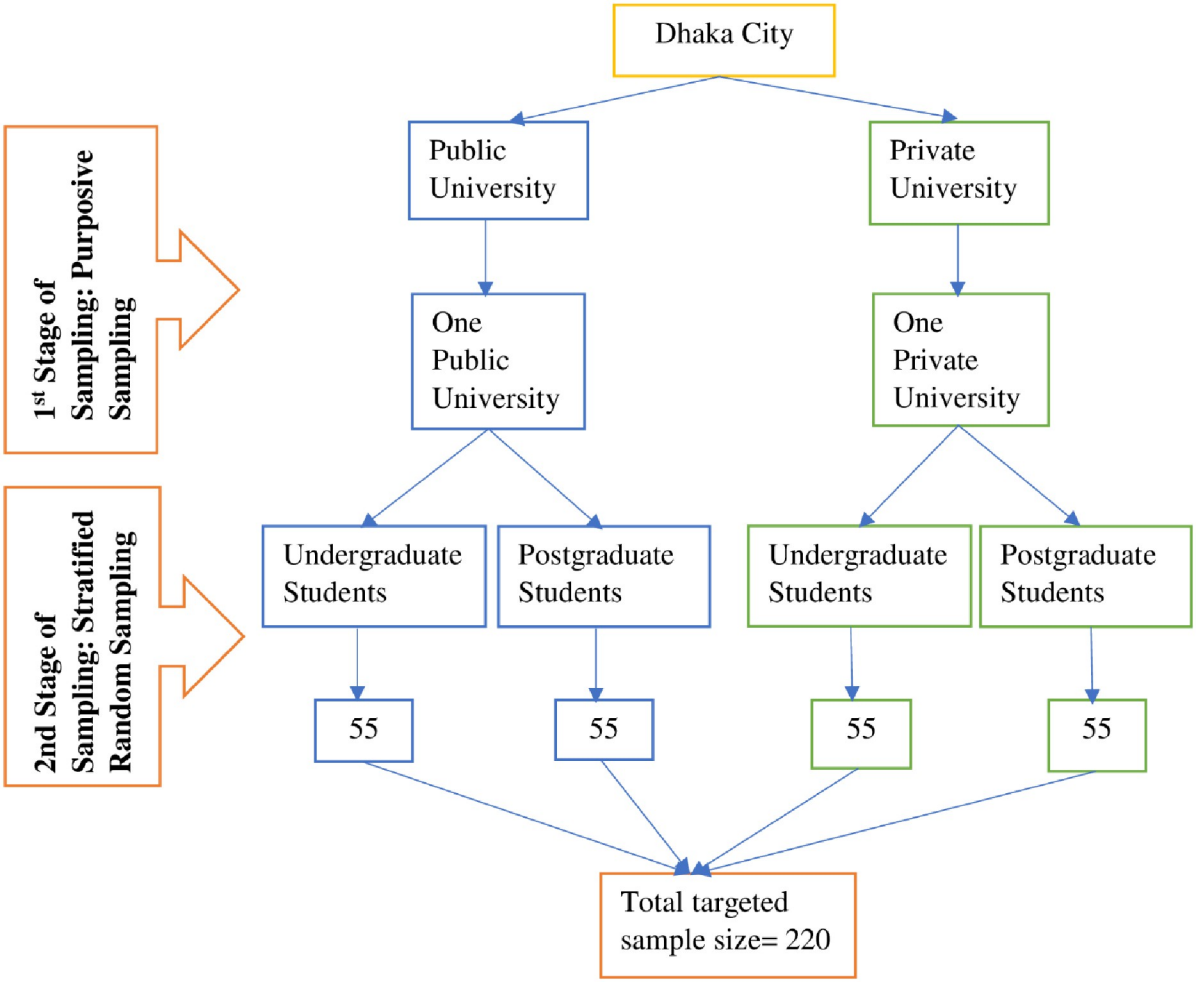
Flowchart of the process of the multi-stage sampling technique.

This cross-sectional study was carried out at two renowned universities in Bangladesh, the University of Dhaka and North South University. Data was collected from March 26 to April 27, 2021. Participants were both undergraduate and postgraduate students. To be included in this study, participants had to be between 18–35 years and be engaged in online learning. Those who could not respond due to severe physical illness were excluded from this study. The study sample size was determined by the given 80% power and 95% CI (0.05 to 1.96), and the prevalence of severe stress from the previous study was 12.8% [32]. With adjustable for 20% refusal, the required sample size was 207. However, 50% of participants were non-responsive throughout the questionnaire pre-testing. As a result, the target sample size was increased to 220 in order to reach the goal. A multistage sampling technique was performed to collect the data (Fig 1). At first, one public and one private university from Dhaka city were chosen purposively. Both universities serve diverse communities, with a higher percentage of graduate students in Bangladesh. For participant selection, a stratified random sampling technique was applied. From the opinion of an expert statistician, at least 55 undergraduate and 55 postgraduate students from both universities (four strata for two universities) were selected.

As per government directives, the campus was closed with classes resumed online; therefore, four student representatives from each university provided each student’s school email for data collection purposes. At first, an invitation was sent with an informed consent paper explaining the purpose of the study and assurance of confidentiality. Participants were asked whether they would consent to participate in the study and if so, an online questionnaire was sent to their school email address. Participants were requested to feel accessible to any queries. The questionnaire was sent to 278 students. Two hundred forty-six students who were interested in participating in this study gave their informed consent, and 222 completed the online questionnaire. The response rate was 79.8%. Lastly, 210 responses were included in the final analysis after a comprehensive examination and review for consistency verification and reduction.

Data was collected using a structured questionnaire prepared in the English language. The baseline socio-demographic variables concerned in this study were age, gender, area of residence, education, and type of university. Participants were asked about their self-income level, having a personal computer/laptop, and availability of internet/wi-fi connection at home; these responses were recorded as ‘yes/no.’ Further, they were asked about their average time spent on online education each day (1–3 hours, 3–5 hours, 5–7 hours), how helpful university has been offering the resources to learn from home (not at all helpful, moderately helpful, very helpful). Moreover, participants self-reported the presence of any mental health illnesses, and it was recorded as (yes, no, and maybe).

A pilot study was conducted to check the research feasibility, where the questionnaire was approached to 20 people anonymously in a separate sample. Afterward, the questionnaire was modified accordingly. The estimated reliability of each scale was checked by Cronbach’s alpha (value 0.941), showing excellent reliability.

### Measures

#### Kessler-10

**Fig 2:**
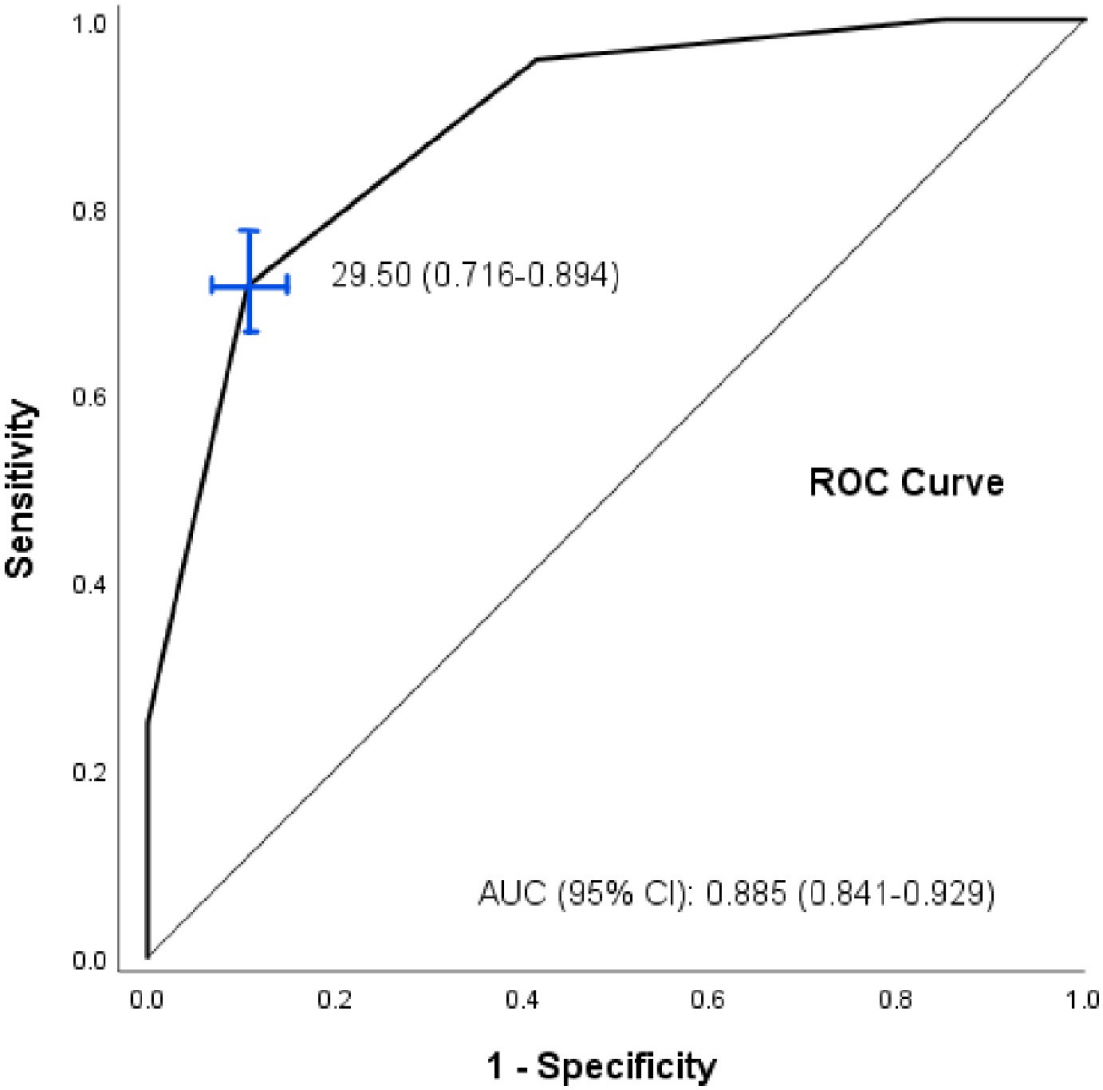
ROC curve; K10 score.

The psychological distress instrument, Kessler-10 (K10), a 10-item self-measured and straightforward questionnaire, was used to measure psychological distress levels. The responses observed participants’ emotional states in the last 4-weeks [33]. Each of these items begins with the proposition: “During the last 30 days, about…” followed by ten items with 5-point Likert response options that range from 1 (none of the time) to 5 (all of the time). A sample item is “…how often did you feel tired out for no good reason?” Based on the stems of the 5-point Likert scale, the scores ranged into four categories (score 10–19 well/no stress, score 20–24 mild stress, score 25–29 moderate stress, score ≥30 severe stress). This study determined the cutoff score of ≥29.5 from each domain, showing a strong predictor of the outcome. The inventory cutoff point was assessed using the receiver operating characteristics (ROC) curve and the area under the ROC curve (AUC). The cutoff point that best discriminated was 29.5; that is, anyone with a score equal to or more than 29.5 was considered severe stress. This value represents a specificity of 71.5% and a sensitivity of 89.4%; the area under the curve (AUC) was found to be [0.885 (CI = 0.841–0.929), *p*-value<0.01)] (Fig 2).

#### Brief COPE

**Fig 3:**
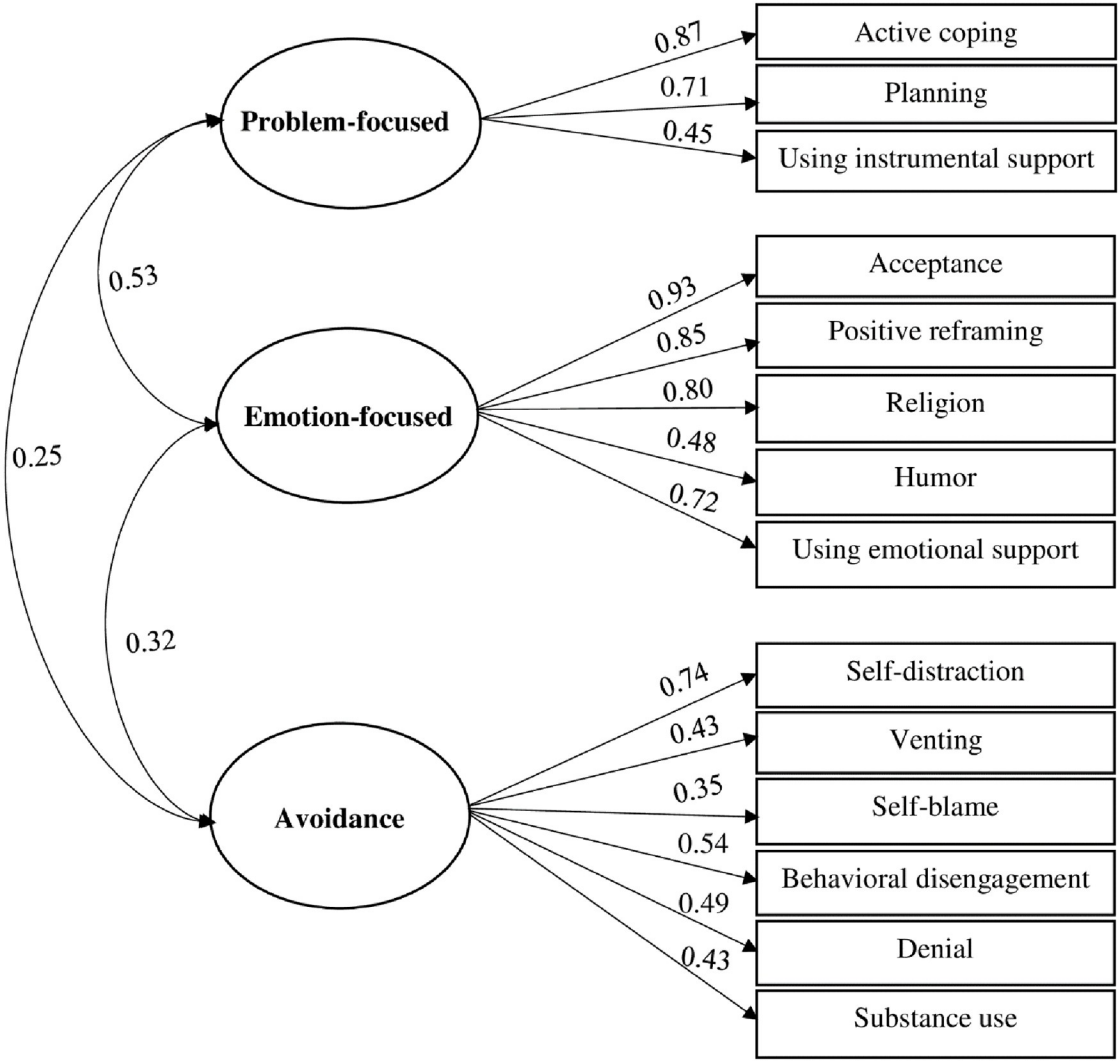
CFA model for the brief-COPE inventory.

A shortened version of the brief-COPE Inventory was used to measure the participants’ coping strategies. COPE Inventory is a multidimensional scale comprised of two items on 14 subscales [29]. These subscales fall into three categories problem-focused, emotion-focused, and avoidance. Brief-COPE Inventory was assessed using a four-item Likert scale ranging from 1 (I usually do not do this at all) to 4 (I usually do this a lot). Each item showed a moderate reliability Cronbach alpha score (0.745–0.783) and a moderate internal consistency of 0.677 (Table 5). Confirmatory factor analysis (CFA) evaluated the construct validity of the hypothesized model [28–30]. Multiple indices were estimated to consider the goodness of the fitted model, the Comparative Fit Index (CFI), the Tucker-Lewis Index (TLI), the Root Mean Square Error of Approximation (RMSEA), the Standardized Root Mean Squared Residual (SRMR) [34]. An acceptable model fit criteria was indicated by RMSEA < 0.05, SRMR < 0.08, TLI, and CFI > 0.90 [35]. The Chi-square test of absolute model fit indicated a fitted model. The overall χ2 for this model was 168.81 (df 74) (*p*<0.001). After including all the hypothetical pathways, the model also demonstrated an adequate fit to the data (CFI = 0.993; TLI = 0.946; RMSEA = 0.078; SRMR = 0.049). The model explained 34% of the variance in brief COPE (Fig 3).

### Statistical analysis

Data were analyzed using the Statistical Package for the Social Sciences (SPSS) software version 25.0. The z-score transformation was performed for each outcome variable to check outliers. Cases with |z| ≥ 3.29 typically indicate potential outliers. By using these criteria, no significant outliers were identified. Descriptive statistics was denoted as means and standard deviations (SD) for continuous variables and number (n) and percentages (%) for categorical variables. Cronbach alpha and intra-class correlation coefficients (ICC) were used to assess the reliability and internal consistency of Kessler-10 (K10) items. Additionally, Pearson (r) correlational coefficients were stated as evidence of concurrent validity. Confirmatory factor analysis was performed to examine the hypothetical model of the brief COPE using SPSS AMOS version 24.0. Pearson’s chi-square test was applied to see the associations between socio-demographic characteristics and psychological distress, where a *p*-value less than 0.05 was considered significant. A multiple linear regression model was generated to calculate the standardized coefficients (Beta) value with a 95% confidence interval. All 14 COPE inventory variables were entered into the model to evaluate the association with the Kessler category. The logistic regression model’s tolerance and variance inflation factors (VIF) were also obtained to check potential multicollinearity. The association between coping strategies outcomes with Kessler categories was conducted using the Spearman correlation test due to its non-parametric distribution.

### Ethical approval

Ethical approval of this study was taken from the Institutional Review Board/Ethical Review Committee of North South University (2021/OR-NSU/IRB/0402). The participants were provided their consent; no incentive was given to anyone, and all data were collected anonymously and would not identify the participants. Participation in this study was entirely voluntary; no one was forced and was allowed to leave while answering the questions. Participants were reassured that all the information collected would be kept strictly confidential and only be used for research purposes.

## Results

Of 210 participants, 105 (50.0%) were male and 105 (50.0%) were female. Two-thirds of them belong to the age group between 18–25 years (67.1%). The majority were residing in urban areas (79.0%). About 53.3% of participants were undergraduate students, and an equal percentage were from public and private universities (50.0%). 60% of them had no self-income level. Most of the participants had a personal computer or laptop (87.6%), and 92.9% had the availability of an internet or wi-fi connection at home. Around 55.2% spent at least 1–3 hours on online education each day. 58.6% of them expressed that their university was moderately helpful in providing the resources needed for online education from home. Only 5.2% of the participants had an existing mental health illness. The prevalence of severe psychological stress was 55.2% (Table 1).

**Table 1:** Socio-demographic variables of the participants & Kessler Psychological Distress Scale (K10). (n = 210).

Variables	Frequency (n)	Percentage (%)
**Age groups, in years**		
18–25	141	67.1
26–30	49	23.3
31–35	20	9.5
**Gender**		
Female	105	50.0
Male	105	50.0
**Area of residence**		
Rural	44	21.0
Urban	166	79.0
**Educational Status**		
Undergraduate	112	53.3
Postgraduate	98	46.7
**Type of University**		
Government	105	50.0
Private	105	50.0
**Self-income level**		
Yes	84	40.0
No	126	60.0
**Having a Personal Computer/Laptop**		
Yes	184	87.6
No	26	12.4
**Availability of Internet/Wi-Fi Connection at Home**		
Yes	195	92.9
No	15	7.1
**Average time spent on online education each day**		
1–3 hours	116	55.2
3–5 hours	59	28.1
5–7 hours	35	16.7
**University has been offering the resources that are helpful to learn from home**		
Not at all helpful	47	22.4
Moderately helpful	123	58.6
Very helpful	40	19.0
**Presence of any mental health illnesses**		
Yes	11	5.2
No	129	61.4
Maybe	70	33.3
**Kessler Psychological Distress Scale (K10)**		
No stress	23	11.0
Mild stress	25	11.9
Moderate stress	46	21.9
Severe stress	116	55.2

The mean Kessler-10 items were [Mean (SD) = 3.21 (1.14)]. Each item showed an excellent reliability Cronbach alpha score (0.901–0.922) and a moderate internal consistency of 0.722 (Table 2).

**Table 2:** The mean, SD and reliability of the Kessler-10 (K10) items.

Kessler-10 items	Mean ± SD	Range	α	ICC
How often did you feel tired out for no good reason?	3.41 ± 0.98	1–5	0.909	
How often did you feel nervous?	3.08 ± 1.09	1–5	0.910	
How often did you feel so nervous that nothing could calm you down?	2.61 ± 1.14	1–5	0.907	
How often did you feel hopeless?	3.18 ± 1.26	1–5	0.904	
How often did you feel restless or fidgety?	3.14 ± 1.09	1–5	0.908	0.722
How often did you feel so restless you could not sit still?	2.64 ± 1.14	1–5	0.908	
How often did you feel depressed?	3.23 ± 1.14	1–5	0.901	
How often did you feel that everything was an effort?	3.30 ± 1.13	1–5	0.922	
How often did you feel so sad that nothing could cheer you up?	2.94 ± 1.17	1–5	0.901	
How often did you feel worthless?	2.96 ± 1.27	1–5	0.904	

Note. SD = standard deviation; α = Cronbach’s alpha; ICC = intraclass correlation coefficient.

The concurrent validity for all the Kessler-10 items was significantly correlated (*p*-value<0.01). Moreover, the normality of each item was checked in terms of its skewness from 0.199 to (-0.265) and kurtosis (-1.031) to (-0.223). The acceptable values of skewness fall (between − 3 and + 3), and kurtosis is appropriate from a range of (− 10 to + 10) recommended for a CFA with maximum likelihood estimation [34] (Table 3).

**Table 3:** Pearson correlations for the Kessler-10 (K10) items.

**Items**	**K1**	**K2**	**K3**	**K4**	**K5**	**K6**	**K7**	**K8**	**K9**	**K10**
K1	--									
K2	0.556**	--								
K3	0.561**	0.684**	--							
K4	0.548**	0.492**	0.570**	--						
K5	0.510**	0.446**	0.479**	0.581**	--					
K6	0.435**	0.476**	0.609**	0.520**	0.653**	--				
K7	0.558**	0.571**	0.545**	0.675**	0.529**	0.566**	--			
K8	0.309**	0.246**	0.284**	0.331**	0.334**	0.265**	0.433**	--		
K9	0.538**	0.509**	0.595**	0.616**	0.559**	0.635**	0.719**	0.476**	--	
K10	0.508**	0.421**	0.496**	0.734**	0.549**	0.536**	0.720**	0.391**	0.684**	--

Note.

**Correlation is significant at the 0.01 level (2-tailed).

The association between psychological distress and socio-demographic variables using Pearson’s chi-square test identified age groups, type of university, self-income level, how helpful the university has been offering the resources from home, and the presence of any mental health illnesses was significantly associated with psychological distress (*p*<0.05). And the results showing, there was a significant relationship between the age group (χ2 (6, N = 210) = 18.330, *p*<0.05), type of university (χ2 (3, N = 210) = 12.079, *p*<0.05), self-income level (χ2 (3, N = 210) = 7.902, *p*<0.05), how helpful university has been offering the resources from home (χ2 (6, N = 210) = 12.661, *p*<0.05) and any existing mental illnesses (χ2 (6, N = 210) = 28.305, *p*<0.05) with psychological distress levels. Therefore, the effect of severe stress was higher among students aged 18–25 years (75.9%). Similarly, public university students showed a raised stress level than private university students (54.3%). A significant proportion of students with no self-income (68.1%) had suffered during online education. Students who expressed receiving moderate resources from their university to continue the classes from home showed a lifted severe stress level than others (57.8%). Further, those with previous mental health illnesses were also significantly associated with psychological distress (7.8%) (Table 4).

**Table 4:** Association between psychological distress and socio-demographic variables.

Variables	Kessler category				χ2 (*p*-value)
	No stress	Mild stress	Moderate stress	Severe stress	
**Age groups, in years**					
18–25	12 (52.2%)	12 (48.0%)	29 (63.0%)	88 (75.9%)	0.005**
26–30	5 (21.7%)	8 (32.0%)	13 (28.3%)	23 (19.8%)	
31–35	6 (26.1%)	5 (20.0%)	4 (8.7%)	5 (4.3%)	
**Gender**					
Female	8 (34.8%)	14 (56.0%)	21 (45.7%)	62 (53.4%)	0.335
Male	15 (65.2%)	11 (44.0%)	25 (54.3%)	54 (46.6%)	
**Area of residence**					
Rural	5 (21.7%)	6 (24.0%)	5 (10.9%)	28 (24.1%)	0.298
Urban	18 (78.3%)	19 (76.0%)	41 (89.1%)	88 (75.9%)	
**Educational Status**					
Undergraduate	11 (47.8%)	11 (44.0%)	25 (54.3%)	65 (56.0%)	0.679
Postgraduate	12 (52.2%)	14 (56.0%)	21 (45.7%)	51 (44.0%)	
**Type of University**					
Government	15 (65.2%)	5 (20.0%)	22 (47.8%)	63 (54.3%)	0.007**
Private	8 (34.8%)	20 (80.0%)	24 (52.2%)	53 (45.7%)	
**Self-income level**					
Yes	13 (56.5%)	13 (52.0%)	21 (45.7%)	37 (31.9%)	0.048*
No	10 (43.5%)	12 (48.0%)	25 (54.3%)	79 (68.1%)	
**Having a Personal Computer/Laptop**					
Yes	21 (91.3%)	23 (92.0%)	40 (87.0%)	100 (86.2%)	0.810
No	2 (8.7%)	2 (8.0%)	6 (13.0%)	16 (13.8%)	
**Availability of Internet/Wi-Fi Connection at Home**					
Yes	22 (95.7%)	25 (100.0%)	41 (89.1%)	107 (92.2%)	0.358
No	1 (4.3%)	0 (0.0%)	5 (10.9%)	9 (7.8%)	
**Average time spent on online education each day**					
1–3 hours	14 (60.9%)	16 (64.0%)	28 (60.9%)	58 (50.0%)	0.213
3–5 hours	5 (21.7%)	8 (32.0%)	14 (30.4%)	32 (27.6%)	
5–7 hours	4 (17.4%)	1 (4.0%)	4 (8.7%)	26 (22.4%)	
**University has been offering the resources that are helpful to learn from home**					
Not at all helpful	4 (17.4%)	0 (0.0%)	14 (30.4%)	29 (25.0%)	0.049*
Moderately helpful	14 (60.9%)	16 (64.0%)	26 (56.5%)	67 (57.8%)	
Very helpful	5 (21.7%)	9 (36.0%)	6 (13.0%)	20 (17.2%)	
**Presence of any mental health illnesses**					
Yes	1 (4.3%)	1 (4.0%)	0 (0.0%)	9 (7.8%)	<0.001**
No	18 (78.3%)	21 (84.0%)	37 (80.4%)	53 (45.7%)	
Maybe	4 (17.4%)	3 (12.0%)	9 (19.6%)	54 (46.6%)	

Note.

**Significant with *p*-value<0.01;

*p-value<0.05.

The distribution of coping strategies based on brief-COPE was shown in (Table 5). Among those, most participants applied problem-focused coping strategies a medium amount to relieve psychological distress, where active coping (41.0%), planning (48.1%), and using instrumental support (33.8%). On the other hand, in emotion-focused coping strategies, participants utilized acceptance (41.9%) and religion (45.2%) a lot to cope with psychological distress. Whereas positive reframing (39.5%), using emotional support (49.5%), and humor (39.0%) involved a medium amount. Moreover, in the avoidance category, self-distraction (35.2%) and venting (37.1%) were used a medium amount by the participants. Self-blame (27.6%) and behavioral disengagement (41.4%) were used a little bit, and the lowest participants applied were denial (37.6%) and substance use (81.4%). The minimum score for coping strategies was 2, and the maximum score was 8. The mean score interpretation showed that the frequently used coping strategies were active coping (5.12±1.53) and acceptance (6.05±1.68). In contrast, substance use (1.27±0.63) was used minimum by the participants.

**Table 5:** Coping strategies based on brief-COPE among the participants. (n = 210).

Coping Strategies	Categories	Frequency (n)	Percentage (%)	Mean ± SD	α
**Problem-focused**					
**Active Coping**	I usually don’t do this at all	9	4.3	5.12 ± 1.53	0.774
	I usually do this a little bit	71	33.8		
	I usually do this a medium amount	86	41.0		
	I usually do this a lot	44	21.0		
**Planning**	I usually don’t do this at all	19	9.0	4.69 ± 1.49	0.760
	I usually do this a little bit	67	31.9		
	I usually do this a medium amount	101	48.1		
	I usually do this a lot	23	11.0		
**Using Instrumental Support**	I usually don’t do this at all	31	14.8	4.93 ± 1.92	0.745
	I usually do this a little bit	62	29.5		
	I usually do this a medium amount	71	33.8		
	I usually do this a lot	46	21.9		
**Emotion-focused**					
**Acceptance**	I usually don’t do this at all	8	3.8	6.05 ± 1.68	0.759
	I usually do this a little bit	28	13.3		
	I usually do this a medium amount	86	41.0		
	I usually do this a lot	88	41.9		
**Positive Reframing**	I usually don’t do this at all	14	6.7	5.72 ± 1.83	0.754
	I usually do this a little bit	40	19.0		
	I usually do this a medium amount	83	39.5		
	I usually do this a lot	73	34.8		
**Religion**	I usually don’t do this at all	21	10.0	5.89 ± 1.95	0.746
	I usually do this a little bit	32	15.2		
	I usually do this a medium amount	62	29.5		
	I usually do this a lot	95	45.2		
**Humor**	I usually don’t do this at all	18	8.6	5.34 ± 1.84	0.761
	I usually do this a little bit	53	25.2		
	I usually do this a medium amount	82	39.0		
	I usually do this a lot	57	27.1		
**Using**	I usually don’t do this at all	8	3.8	5.50 ± 1.57	0.746
**Emotional Support**	I usually do this a little bit	43	20.5		
	I usually do this a medium amount	104	49.5		
	I usually do this a lot	55	26.2		
**Avoidance**					
**Self-Distraction**	I usually don’t do this at all	17	8.1	5.52 ± 1.79	0.749
	I usually do this a little bit	46	21.9		
	I usually do this a medium amount	74	35.2		
	I usually do this a lot	73	34.8		
**Venting**	I usually don’t do this at all	22	10.5	4.83 ± 1.74	0.752
	I usually do this a little bit	70	33.3		
	I usually do this a medium amount	78	37.1		
	I usually do this a lot	40	19.0		
**Self-Blame**	I usually don’t do this at all	49	23.3	2.48 ± 1.08	0.760
	I usually do this a little bit	58	27.6		
	I usually do this a medium amount	56	26.7		
	I usually do this a lot	47	22.4		
**Behavioral Disengagement**	I usually don’t do this at all	50	23.8	2.21 ± 0.92	0.770
	I usually do this a little bit	87	41.4		
	I usually do this a medium amount	52	24.8		
	I usually do this a lot	21	10.0		
**Denial**	I usually don’t do this at all	79	37.6	1.97 ± 0.94	0.782
	I usually do this a little bit	77	36.7		
	I usually do this a medium amount	37	17.6		
	I usually do this a lot	17	8.1		
**Substance Use**	I usually don’t do this at all	171	81.4	1.27 ± 0.63	0.783
	I usually do this a little bit	25	11.9		
	I usually do this a medium amount	11	5.2		
	I usually do this a lot	3	1.4		

Note. COPE = coping orientation to problems experienced; SD = standard deviation; α = Cronbach’s alpha; ICC = intraclass correlation coefficient.

Based on the previous studies, a multiple linear regression model was employed to evaluate whether the category of stressor influences frequently used coping strategies [36–38]. The stepwise method was used to include all 14 variables of brief COPE inventory in the multivariate analysis. The results demonstrate that the main effect was small yet significant, F (14, 195) = 5.697, *p*<0.001, showing that people used some coping strategies relatively more than others. The model provided a good fit and could explain 29.0% of the psychological distress on K10 scores (R-square = 0.290). Among all the categories, active coping, using emotional support, and self-blame were significantly associated (*p*-value<0.01)). The unstandardized coefficient (B) for active coping (-0.198); 95% CI (-0.363) to (-0.033), using emotional support (-0.290); 95% CI (-0.472) to (-0.109) and self-blame 0.234; 95% CI (0.101 to 0.367). The standardized coefficients (Beta) values for active coping, using emotional support, and self-blame were (-0.157), (-0.221), and 0.245, respectively. It indicates that these variables had more influence on the Kessler category. The higher value indicates more influence on the dependent variable. In this study, self-blame had a greater influence on explaining the Kessler category than the other two variables (Table 6).

**Table 6:** Association between Kessler category and variables of COPE inventory^a^.

Variables	Unstandardized Coefficients B	Standardized Coefficients Beta	*P* − value^b^	95% CI	
				LCL	UCL
**Active Coping**	-0.198	-0.157	0.019**	-0.363	-0.033
**Planning**	0.060	0.046	0.486	-0.109	0.229
**Using Instrumental Support**	0.088	0.083	0.220	-0.053	0.228
**Acceptance**	-0.146	-0.115	0.122	-0.331	0.039
**Positive Reframing**	-0.048	-0.042	0.603	-0.231	0.134
**Religion**	-0.033	-0.032	0.646	-0.176	0.109
**Humor**	0.102	0.091	0.164	-0.042	0.246
**Using Emotional Support**	-0.290	-0.221	0.002**	-0.472	-0.109
**Self-Distraction**	-0.003	-0.003	0.969	-0.153	0.147
**Venting**	0.064	0.056	0.413	-0.090	0.217
**Self-Blame**	0.234	0.245	0.001**	0.101	0.367
**Behavioral Disengagement**	0.087	0.077	0.283	-0.072	0.246
**Denial**	0.151	0.137	0.063	-0.008	0.309
**Substance Use**	0.092	0.056	0.369	-0.110	0.294

Note.

**Significant with *p*-value<0.01.

^a^. Dependent Variable: Kessler category.

^b^. F (14, 195) = 5.697, *p*<0.001, R-square = 0.290.

The association between coping strategies outcome with Kessler categories using Spearman correlation due to non-parametric distribution was displayed in (Table 7). The results indicated that participants who applied emotion-focused coping strategies had a significant negative but weak correlation with psychological distress *r* (208) = (−0.146), *p*<0.05. Furthermore, participants who applied avoidance as their coping strategies had a significant positive and moderate correlation with psychological distress *r* (208) = 0.348, *p*<0.01.

**Table 7:** Spearman correlation between coping strategies and psychological distress.

Coping Strategies	Kessler Category	
	*r*_*s*_ (-1.0 to +1.0)	*p*-value
**Problem-focused**	-0.090	0.192
**Emotion-focused**	-0.146	0.035*
**Avoidance**	0.348	<0.001**

Note.

*Correlation is significant at the 0.05 level (2-tailed) &

**Correlation is significant at the 0.01 level (2-tailed).

## Discussion

### Main findings

The COVID-19 pandemic forced institutional activities to shift from the conventional format to that of online learning. This sudden alteration to daily life induced higher anxiety, depression, and stress rates in students. A consideration of the benefits of coping strategy resources is necessary for students experiencing stressful situations. This study examined the association between psychological distress and coping strategies among students engaged in online learning. The findings of this study showed that Bangladeshi university students’ psychological distress levels were heightened during the pandemic. In addition, the results also showed that emotion-focused and avoidance coping strategies were effectively utilized by students experiencing stress for a better outcome.

### Comparisons with other studies

The severe psychological distress prevalence rate was 55.2% among Bangladeshi university students engaged in online learning. Although this may appear daunting, such experiences are quite common. A recent study conducted at Texas A&M University, USA, found that 71% of students reported higher stress and anxiety during COVID-19 [39]. Similarly, the prevalence of anxiety and stress was 32.9% and 14.6%, respectively, in medical school graduates in China [40]. Perceived stress among University of Nizwa students in Oman reported that 82.5% were experiencing medium stress, while 14.4% experienced raised stress during COVID-19 e-learning [41]. A sample of 420 primary and secondary school students in Palestine showed that 35.7% experienced moderate to severe stress levels [42]. University students are primarily acknowledged as a vulnerable population; therefore, the radical shift in education affects students regardless of their socio-economic status. A cross-national study conducted among university students in nine countries discovered that high stress, depression, and anxiety prevalence was 61.30%, 40.3%, and 30%, respectively [43]. The prevalence of depressive symptoms, suicidal ideation, and perceived stress was reportedly higher among university students in the humanities field in Italy, where 87.7% reported moderate or severe stress [44].

Among all socio-demographic groups, the younger age group of 18–25 years experienced the most distress. Similar results were seen in studies from Malaysia and Switzerland, where psychological well-being and distress-related factors were significant in the 20–25 years age group and in female students [45,46]. Concerning psychological distress severity, another study in Jordan reported that 54.9% of students found no motivation for distance learning, which was very stressful for those 18–22 years of age [47]. Self-reported stress levels were also higher among high school students, resembling statistics of students in primary school in China [48]. In addition, public university students tended to suffer more (54.3%) than private university students. This appeared particularly evident in Bangladesh, as they lacked the facilities to engage in online learning [18,49]. Following the suspension of conventional classes, public university students experienced widespread panic and increased anxiety and stress during the COVID-19 outbreak. A recent survey conducted by the psychology department of six states in the USA showed that only 35% of students had taken one or more courses online before the pandemic. Therefore, anxiety and distress during COVID-19 appear to show a solid correlation to unfamiliarity with online learning [50].

Additionally, 68.1% of participants with no self-income reported being prone to severe stress. According to a study from Australia, female students suffered medium to high psychological distress levels because of employment loss during the pandemic period [51]. From Adams et al., the study conducted among Midwestern university students found that approximately 38% depended on family income as they did not have any self-income source [52]. Another cross-sectional study from the University of Thessaly, Greece, reported that students with financial dissatisfaction were most likely to experience severe psychological distress among all factors. [53]. The 2017 National Health Interview Survey (NHIS) sample showed that U.S. adults experiencing financial worries and food and healthcare insecurity were more likely to face psychological distress [54]. In North America, young adults, females, and low-income groups were the risk factors for pandemic-related stressors during COVID-19 [55]. Similarly, the unemployment rate and loneliness increased the likelihood of experiencing adverse mental health outcomes among German female participants [56].

Moreover, the results of this study also found that participants who received moderate resources from the university to continue online learning did not benefit significantly from them, with 57.8% of students reporting severe stress. A study conducted in New Jersey, USA, showed increased concern for academic performance among students engaged in online learning. A lack of social interaction affected their ability to concentrate and caused a rise in stress levels [57]. Results from a cross-sectional study in Munich also showed that severe psychological distress was associated with lower life satisfaction, inadequate social support, lack of social interaction, and worries about financial difficulties [58].

Further, this study found that 7.8% of participants with previous mental illnesses experienced significant psychological distress. Although it is expected that preexisting mental health conditions correlated to raised psychological distress during the pandemic, this was not always the case. A study in Canada showed that students without preexisting mental health concerns were more likely to suffer a decline in mental health [59]. Mboya et al. discovered that a family history of mental illness was significant in predicting future psychological distress [60]. A strong parent-child relationship, regular interactions, a problem-solving mentality, and regular communication have been shown to benefit a child’s mental health. Similarly, a family history of psychiatric disorders increased the likelihood of experiencing depressive symptoms among Italian students, and female university graduates who were already consulting a psychologist or psychiatrist were positively associated with suffering psychological distress [44].

The multivariate analysis of the present study demonstrated that active coping, using emotional support, and self-blame were more commonly used by Bangladeshi university students to overcome the stressful circumstances of online learning during the COVID-19 pandemic. Comparable results were seen in an exploratory study in Poland, where female and postgraduate students used various strategies to manage stress, including active coping, acceptance, and positive reframing [61]. Another study conducted among U.S. Mexican college students found that active coping and avoidance strategies were related to coping with stress [62]. From Ismail et al., Malaysian medical college interns found problem-focused and avoidance coping strategies helpful in coping with stress [45]. In contrast, seeking social assistance was the least favored option among Pakistani students, with most students choosing emotion-focused and problem-focused coping strategies to lower psychological distress [63]. As a result of being forced to stay at home, undergraduate health science students at Jimma University in Ethiopia employed active coping, planning, and positive reframing in their everyday lives during COVID-19 [64]. Acceptance, planning, and seeking emotional support were also the most commonly employed stress management approaches among Polish students [65].

Although there has been some debate about whether avoidance coping strategies have benefits, research has shown that while they may help reduce short-term stress, they are generally considered unhealthy [66]. An increased prevalence rate of depression, anxiety, and stress symptoms was seen in Brazilian undergraduate female students aged 18–24 years old; these students were more likely to use avoidance coping strategies such as self-blame and substance use [67]. Another study conducted in the United Kingdom during the COVID-19 pandemic discovered that participants with previous mental health conditions were more likely to use avoidant coping and social support; in contrast, others preferred supporting strategies such as problem-focused strategies [68]. Further, Vietnamese research of public health and preventive medicine students discovered that individuals experiencing extreme stress were more likely to use avoidance coping techniques [69]. In contrast, substance use was least common among students from Saudi Arabia and Egypt, where coping with religious activities and spiritual beliefs in severe stressful conditions proved beneficial [32,70].

A study by the University of Aberdeen, Scotland, showed that students who self-harmed found coping strategies to be an effective way of dealing with their emotions and behaviors [71]. Among university students, existing mental health difficulties, such as stress and depressive symptoms, are exacerbated by a lack of social support. A Malaysian study found a connection between perceived social support and psychological distress among university graduates [72]. Similar results were seen in Mexican American and Latin American undergraduate students, where students with available perceived social support displayed lower psychological distress than students who did not seek such support [73]. Research showed that Latino College students willing to seek psychological help experienced better health outcomes [74]. University students’ most common coping techniques in the Philippines were resource management, help-seeking, technological skill enhancement, time managing, and learning environment control [75].

This study was conducted during the early stages of the pandemic, surveying Bangladeshi university students’ mental health; it was aimed at identifying strategies that students found helpful in their daily activities. Due to the wealth of information these studies provide, coping strategies and adaptive responses must receive more attention and discussion, as they play an immense role in helping students cope with crises such as COVID-19.

## Implications of this study

A significant number of students worldwide encountered similar psychological distress throughout this pandemic. Unlike American and European countries, Bangladesh follows a tradition of classroom-centered redundant pedagogy. Therefore, online learning methods are less frequently seen as a tool for delivering lessons. This global pandemic produced immense mental pressure for students who suffered from a lack of knowledge of self-directed online delivery of instruction. The results of this study provide several suggestions for university stakeholders concerning purposeful education. Different adaptive or non-adaptive strategies, such as active behavioral coping, have shown positive outcomes when used by students.

Stress can significantly impact a student’s academic performance and personal life. University students with disrupted daily activities struggled immensely when forced to experiment with distance learning during COVID-19. The lack of interaction proved that students needed help to overcome this situation. Routine screening will provide every student with emotional, social, and psychological support. Whether public or private, each institution should have one mental health counseling office for students to receive help from experts whenever they need it. The student health and counseling center can arrange monthly stress management workshops focusing on coping techniques and relaxation exercises. Also, universities should have an option for virtual meetings where students can share their thoughts in a private session that might help identify the risk groups. The student association counselor can also provide social support. Higher degrees of perceived social support can impact students’ coping strategies, resulting in decreased stress and anxiety. Social support is directly connected to self-esteem; therefore, by staying in touch, returning emails, and talking on the phone, students can improve their emotional wellness.

This study also found that students with no self-income were most likely to have experience severe psychological stress. This indicates that universities should ensure students’ financial stability by offering academic scholarships and student loans. Administrative support provided by student ambassadors can also guide new students, as well as offer other students part-time employment. Students in this study also expressed that the resources provided by their universities were inadequate, causing mental pressure. Therefore, university administration could best serve their scholars by providing online support like internet bandwidth to ensure students have uninterrupted lessons. Alternatively, self-directed learning among students can offer a less stressful environment and improve students’ options, self-confidence, and motivation.

The current study findings emphasize the importance of coping skills and the crucial need for mental health education programs to be integrated into the university curriculum. Students’ task-oriented coping strategies are an indication of their problem-solving abilities. Most institutions teach major disciplines and rely on their students to learn good decision-making skills and tenacity along the route. However, in recent months, students around the world have faced challenges for which they were unprepared. Incorporating the COPE-Resilience program into the first-year university curriculum could benefit students in advancing social-emotional skills in a pleasant learning environment.

## Limitations and recommendations

The findings of the current study should be weighed against several drawbacks. Because this study was cross-sectional, determining the causation of the observed relationships was difficult. Despite the fact that the analytical model was built on a widely accepted theoretical framework, the potential of reversed causality cannot be ruled out. Future research should employ a longitudinal study design. Because the study sample size was limited, future studies should include more participants to evaluate psychological distress levels among students. Additionally, future research could also benefit from exploring coping strategies’ effectiveness in diverse populations and cultural settings. A strength of this study was using a validated scale of Kessler-10 items. Each component showed excellent reliability and internal consistency of Kessler-10 items, and all items were intercorrelated. Concerning brief-COPE items, there was a criticism of its subscales and structure. Nonetheless, the findings of this study provide accepted outcomes and practical application of COPE inventory variables in managing stressful situations.

## Conclusion

This study aimed to ascertain the association between psychological distress and coping strategies among students engaged in online learning. Along with many socio-demographic variables, the study found that younger age groups, public university students, students with no self-income level, moderate help from universities in offering the resources from home, and students with previous mental health illnesses were significantly associated with psychological distress. The present study also found that active coping, using emotional support, and self-blame significantly influenced managing psychological distress. Therefore, every institution must address the mental health necessities of its students. Regular screening and support from experts by counseling would improve students’ psychological health.
